# Identification of SNPs in the second intron of *IGF2BP1* and their Association with growth traits in Nanjiang Yellow goat

**DOI:** 10.1080/10495398.2025.2461176

**Published:** 2025-02-17

**Authors:** Shuheng Chen, Liang Xu, Junchen Leng, Zitong Chen, Yu. Chen, Li. Li, Hongping Zhang, Mingzhou Li, Jiaxue Cao

**Affiliations:** a Ministry of Agriculture and Rural Affairs, College of Animal Science and Technology, Key Laboratory of Livestock and Poultry Multiomics, Sichuan Agricultural University, Chengdu, China; b Xinjiang Yili Prefecture Animal Husbandry Station, Yining, China; c Sichuan Nanjiang Yellow goat Breeding Farm, Nanjiang, China

**Keywords:** *IGF2BP1* gene, association analysis, Nanjiang Yellow goat, growth traits, SNP, dual luciferase

## Abstract

Insulin-like Growth Factor 2 mRNA-binding Protein 1 (*IGF2BP1*) is a candidate gene of significant interest for modulating economically important traits in livestock and poultry. The second intron of *IGF2BP1* has been implicated in growth-related traits, though its precise mechanistic role remains elusive. Initial resequencing analyses in our laboratory indicated strong selective pressures on the *IGF2BP1* genomic region, prompting the selection and identification of several single nucleotide polymorphisms (SNPs). Seven SNPs were mapped to the conserved region of the second intron, necessitating further investigation into their functional relevance and association with growth traits. In this study, 348 Nanjiang Yellow goats were analyzed, and the association analysis via the GLM program in SAS 9.4 identified five SNPs significantly correlated with growth traits. Notably, rs652062749(A > G) emerged as a critical locus influencing later-stage growth traits. Furthermore, strong linkage disequilibrium was observed among three SNPs, with the rs638185407 (T > A) variant markedly enhancing luciferase activity in H293T cells. Combination genotypes TTAACT, TTCCCC, and ATCACT were identified as superior for growth traits, offering theoretical insights for genetic co-breeding. This study underscores the potential utility of *IGF2BP1* as a functional genetic marker in Nanjiang Yellow goat breeding programs.

## 1. Introduction

Goats are one of the earliest domesticated livestock species and have a wide variety of breeds, which can be classified according to the type of products they supply. These include fiber goats,^1^ dairy goats,^2^ and meat goats.^3^ According to the FAOSTAT database, in conjunction with the increasing consumer demand for goat meat in the international market, the world’s major countries are continuously modifying their production structures, placing greater and greater emphasis on meat goat production.^4^ However, according to the FAOSTAT database, China’s meat goat carcass weight level still lags behind that of some developed countries in the global meat goat industry. Therefore, it is imperative to select and breed Chinese superior meat goat breeds to enhance the growth traits of Chinese meat goats. Nanjiang Yellow goat is the first meat goat breed independently bred in China, which has been carefully cultivated for 40 years, and has various characteristics of genetic stability, suitable germplasm, strong adaptability, and fast growth and development. In comparison to the majority of local Chinese goat breeds, the Nanjiang Yellow goat exhibits a distinctive feature in its rapid growth and development.^5^ However, the underlying mechanisms driving this phenomenon remain elusive and need further in-depth investigation.

Insulin-like Growth Factor 2 mRNA binding Protein 1 (*IGF2BP1*) belongs to the Insulin-like Growth Factor 2 mRNA binding Proteins (*IGF2BP*s) gene family. This gene family plays a crucial role in cell proliferation and growth in normal tissues, tumor cell adhesion, apoptosis, migration, and invasion. *IGF2BP1* plays a pivotal role in the regulation of cancerous diseases, embryonic and organ growth, and development, both through its own action and through interactions with other genes. Additionally, it serves as a crucial reference point for studies related to livestock and poultry growth and development.^6–9^ As a vital candidate gene affecting animal growth and development, its polymorphisms have been analyzed in association with growth traits in several livestock and poultry species. In chicken’s whole genome sequencing studies, strong selection was found near *IGF2BP1*, associated with breast muscle yield and carcass traits in chickens. Therefore, it was hypothesized that *IGF2BP1* was proposed to be the primary effector gene regulating carcass traits.^10^ The *IGF2BP1* gene is also the primary effector gene for body size enlargement in Peking ducks, with a mutation at the upstream 148 kb locus. This resulted in the continued high expression of the *IGF2BP1* gene after emergence from the carcass, which led to an increase in the feed utilization efficiency of Pekin ducks and, ultimately, their body size.^11^

The second intron of this gene represents a particularly relevant area for further investigation, as it contains the potential for single nucleotide polymorphism (SNP) associated with growth traits in several livestock species. A study on Chaohu ducks has shown the deletion in intron 2 of the IGF2BP1 gene had a significant effect on the intramuscular fat content in the muscle of the male ducks.^12^ The study of polymorphism of the IGF2BP1 gene in Shaanbei white cashmere goats revealed the presence of two polymorphic sites associated with body slant length, chest depth, chest width, and tube circumference. The highest body weight of Shaanbei white cashmere goats was found when an insertion of an InDel of 15 bp in the 2nd intron and an InDel of 5 bp in the 3′-UTR region was observed purely.^13^ A study for the correlation analyses of growth traits in sheep showed that the 15 bp InDel of IGF2BP1 intron 2 was significantly correlated with growth traits.^14^ Therefore, it is significant to research the relationship between the *IGF2BP1* second intron SNPs and the growth performance of Nanjiang yellow goat.

Previous studies have shown that *IGF2BP1* expression is highest in goats during the pre-rapid muscle fiber growth phase (45 days of the embryonic stage).^15^ This suggests that *IGF2BP1* may be an essential gene affecting the increase in muscle fibers. After overexpressing the *IGF2BP1* gene in Skeletal Muscle Satellite Cells (MuSCs), the number of myoblasts increased significantly, confirming that *IGF2BP1* promotes the proliferation of goat myoblasts.^16^ In the pre-laboratory resequencing study of six goat breeds, we discovered that the *IGF2BP1* gene region 37,175,001–37,275,000 on chromosome 19 of the Meigu goat was strongly selected.^17^ Upon comparison of the goat *IGF2BP1* gene with its human counterpart region through the Ensembl online database, we identified the presence of promoter or enhancer cis-regulatory elements in several regions.

Thus, based on the resequencing results, this study verified and analyzed a total of seven SNPs in the conserved region on the second intron of the *IGF2BP1* gene of the Nanjiang Yellow goat. Subsequently, cellular-level validation was conducted to resolve the functions of the SNPs preliminarily. The objective of this study is to establish a theoretical foundation for the future selection of molecular genetic markers in Nanjiang Yellow goats. This was achieved by selecting functional regions that include regulatory elements, and exploring the correlation between SNPs within the second intron of *IGF2BP1* and growth traits across various developmental periods.

## 2. Materials and methods

### 2.1. Animals and sample collection

The experiment utilized a population of Nanjiang Yellow goats (n = 348) without recording any pedigree information and was exclusively sourced from the Nanjiang Yellow goat stock farm. Throughout the study, all of these goats were subjected to random selection, uniform management practices, and consistent environmental conditions. Their dietary nutrient levels, necessary for growth, were maintained through grazing and suitable supplementary feeding. Each test goat had 1.5 mL of whole blood collected through jugular vein puncture, which was then anticoagulated using heparin sodium and stored at −20 °C for subsequent genomic DNA extraction.

### 2.2. MuSCs isolation and Identification

We successfully isolated MuSCs from the LD muscle of a 1-day-old male kid.^18^ Then, we utilized antibodies against myogenic marker genes MyHC and Pax7 (Santa Cruz, CA, USA) for immunofluorescence. The isolated MuSCs were then preserved in liquid nitrogen tanks for future use.

### 2.3. Cell culture and transfection

MuSCs were cultured at 37 °C with 5% CO_2_ in a growth medium consisting of 1% penicillin-streptomycin (Invitrogen, NY, USA), 10% fetal bovine serum (FBS; Gibco, NY, USA), 89% Dulbecco’s modified eagle medium (DMEM) as well.^19^ Plasmid transfection into MuSCs was performed by Lipofectamine 3000 (Life Technologies, Carlsbad, CA, USA).

### 2.4. Dual-Luciferase reporter assays

The fragments containing three SNPs (rs638185407(T > A), rs640683953(A > C), rs654358008(G > C)) were respectively inserted into the PGL4.23 empty vector (Knp I and Hind III were restriction sites; Supplementary Figure S1). Mutant-type (MUT, the minor) and Wild-type (WT, the major) plasmids were transfected into MuSCs and H293T, respectively. Besides, Luciferase activity was measured using the dual-luciferase reporter kit (Solarbio, Beijing, China), with primers for restriction enzyme digestion listed in Supplementary Table S1.

### 2.5. Extraction of genomic DNA and DNA Quality Testing

Genomic DNA extraction involves using the standard blood genome extraction kit (Tiangen, Beijing, China). DNA quality was assessed via 1.5% agarose gel electrophoresis and ultraviolet imaging with a BIO-RAD ChemDOC XRS gel image analyzer. Analysis was performed by Quality One 4.6.2. The DNA purity and concentration were measured by a nucleic acid protein detector (BIO-RAD, Hercules, CA, USA), and suitable samples were subsequently stored at −20 °C for further testing. Gel electrophoresis results are depicted in Supplementary Figure S2.

### 2.6. PCR amplification and sequencing

Each SNP and the corresponding flanking sequences were obtained from the Ensembl based on position. Primers were designed by Primer Premier 6.0 and synthesized by Sangon (Shanghai, China) based on these sequences. Additionally, birth records from the Nanjiang Yellow goat breeding farm were reviewed and 20 DNA samples extracted from a total of 348 goats were selected. These samples were diluted to a concentration of 20 ng/µL, and 2 µL of DNA from each sample was mixed entirely. This pooled DNA sample is regarded as the template for PCR amplification. Subsequently, PCR products were sequenced bi-directionally by Shanghai Sangon (Shanghai, China), with SNP verification using SnapGene 6.0.2 by comparing results to the reference genome sequence. Primer details are provided in Supplementary Table S2.

### 2.7. MassARRAY genotyping

348 Nanjiang Yellow goats in total were genotyped by the Sequenom MassARRAY genotyping method (Supplementary Table S3). Information on 7 SNPs and 100 bp sequences upstream and downstream of each SNP was retrieved from the Ensembl database. Following this, fragments containing the SNPs were amplified using the single-base primer extension method and analyzed via MALDI-TOF to distinguish genotypes based on molecular weight. All genomic DNA samples from blood were sent to Fuyu Biotechnology (Beijing, China) for genotyping purposes.

### 2.8. Growth trait measurements

The birth weight, body weight (BW), body height (BH), body length (BL), and chest circumference (CC) of these 348 Nanjiang Yellow goats were assessed by established techniques at 4, 6, 12, and 18 months of age. Birth weight was defined as the weight recorded within 12 hours of birth. BW was determined by taking three measurements with a steelyard and calculating the average. BH represented the vertical distance from the highest point of the girth to the ground. Moreover, BL was measured as the straight distance from the leading edge of the scapula to the hip. Besides, CC indicated the length around the chest, which measured from the rear end of the shoulder blade.

### 2.9. Bioinformatics and data analysis

Jaspar 2022 (http://jaspar.genereg.net/) was employed to predict alterations in binding sites for transcription factors at the corresponding mutant loci within non-coding regions. Analysis of linkage disequilibrium and Hardy–Weinberg equilibrium among these SNPs was conducted using Haploview4.2. Associations of SNPs and Combination genotypes with Growth Traits in goats were analyzed using SAS 9.4, as well as genotypic effects. The General Linear Model (GLM) within SAS 9.4 was utilized to formulate the model.

1. Single genotype effect analysis:

The model used for analysis was Y_ijmn_ = µ +D_i_ + G_j_ +P_m_ + S_n_ + e_ijmn_, where Y_ijmn_ represents the phenotypic observations; µ is the mean value; D_i_ is the fixed effect of date of birth (year and month); G_j_ is the fixed effect of genotype; P_m_ is the fixed effect of place, where the place refers to several sites of the Nanjiang Yellow Goat Original Breeding Farm; S_n_ is the fixed effect of sex; and e_ijmn_ is random error term. All values are expressed as mean ± standard deviation, with results considered statistically significant at p < 0.05.

1. Combination genotype effect analysis:

Based on the model we constructed above, genotype effects were added.

## 3. Results

### 3.1. Polymorphism of the second intron gene of IGF2BP1 in Nanjiang Yellow goat

**Figure 1.**
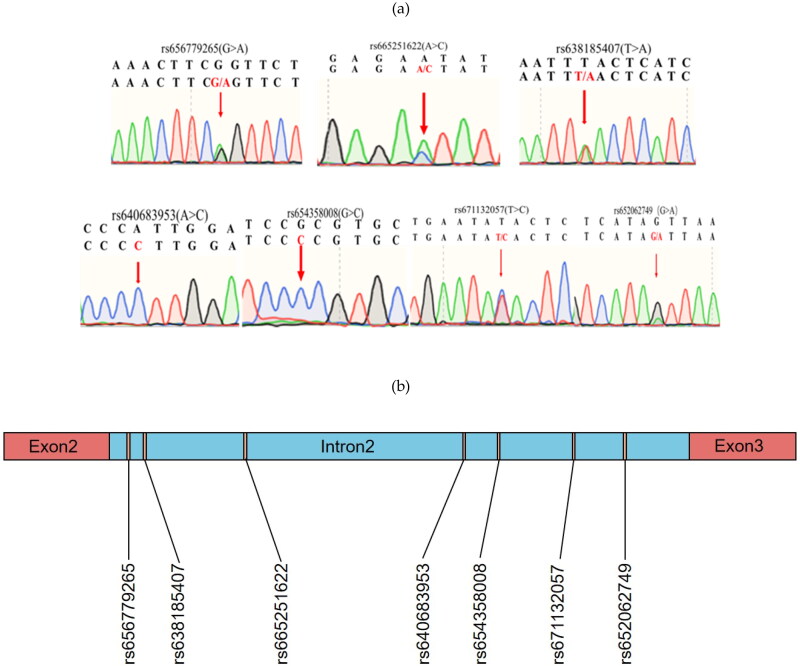
(a) The sequencing results of the seven SNPs from the pooled samples were located in the second intron of *IGF2BP1* gene. In the figure, the base marked by an arrow indicates the genomic locus of each corresponding SNP. And a double peak at these loci indicates the existence of the SNP in the Nanjiang Yellow goat population. The first base sequence is the reference sequence from the database which can be considered as the wild type. And the second base sequence is the sequence obtained by sanger sequencing where the different types can be considered as the mutant type. Besides, the green, red, blue, and black lines represent A, T, C, and G, respectively. (b) Seven selected SNPs in the second intron of *IGF2BP1*. In the figure, the red, blue, and orange parts represent the exon, intron, and selected sites, respectively.

A total of seven SNPs were identified in the second intron of *IGF2BP1* in the experimental population by mixed-pool sequencing. Among them, rs656779265 (G > A), rs638185407 (T > A), rs665251622 (A > C), rs671132057 (T > C) and rs652062749 (G > A) were detected with polymorphisms, while both rs640683953 (A > C) and rs654358008 (G > C) were only found the C type (Figure 1).

### 3.2. Mass spectrometric typing of seven SNPs in the second intron of IGF2BP1 in Nanjiang Yellow goat

**Figure 2.**
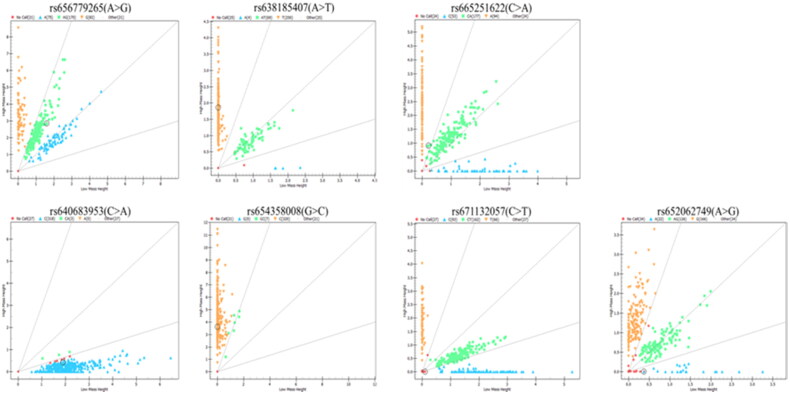
Mass spectrometry results.

The mass spectrometry was utilized to test the genetic distribution of seven SNPs in 348 goats. The result demonstrated the presence of all seven SNPs within the goat population (Figure 2). According to the results of population genetic parameter analysis of seven SNPs in the second intron of *IGF2BP1* in Nanjiang Yellow goat, the genotype frequencies and allele frequencies of the seven SNPs are shown in Table 1 through statistics analysis.

**Table 1.** Genetic parameters and Hardy–Weinberg equilibrium analysis of 7 SNP populations in the second intron of *IGF2BP1* gene.

Locus	Genotype	Genotype frequency	Allele frequency	Ho^1^	He^2^	Ne^3^	H-W^4^	PIC^5^
rs656779265 (G > A)	AA	0.23	0.49 (A)	0.5	0.5	2	0.52	0.37
	AG	0.52	0.51 (G)					
	GG	0.25						
rs638185407 (T > A)	AA	0.01	0.12 (A)	0.79	0.21	1.27	1	0.19
	AT	0.21	0.88 (T)					
	TT	0.77						
rs665251622 (A > C)	AA	0.29	0.56 (A)	0.51	0.49	1.97	0.06	0.37
	CA	0.55	0.44 (C)					
	CC	0.16						
rs671132057 (T > C)	CC	0.29	0.54 (C)	0.5	0.5	1.99	0.88	0.37
	CT	0.5	0.46 (T)					
	TT	0.21						
rs652062749 (G > A)	AA	0.07	0.27 (A)	0.61	0.39	1.65	0.39	0.32
	AG	0.4	0.73 (G)					
	GG	0.53						
rs640683953 (A > C)	CC	0.99	0.99 (C)	0.99	0.01	1.01	1	0.01
	CA	0.01	0.01 (A)					
rs654358008 (G > C)	CC	0.98	0.99 (C)	0.98	0.02	1.02	1	0.02
	GC	0.02	0.01 (G)					

^1,2,3,4,5^ represent homozygosity, heterozygosity, effective number of alleles, the p-value of Hardy-Weinberg equilibrium, and polymorphism information, respectively. And p > 0.05 indicates the locus is under Hardy–Weinberg equilibrium.

In the test population, rs640683953 (A > C) had the highest degree of purity at 0.99, followed by rs654358008 (G > C) at 0.98. rs656779265 (G > A) and rs671132057 (T > C) had the lowest degree of purity at 0.50. Four of the SNPs (rs656779265 (G > A), rs665251622 (A > C), rs671132057 (T > C), and rs652062749 (G > A)) showed moderate polymorphism (0.25 < PIC < 0. 5), and three SNPs (rs638185407 (T > A), rs640683953 (A > C), and rs654358008 (G > C)), had a low degree of polymorphism (PIC < 0.25). The Hardy-Weinberg equilibrium test indicated that all SNPs conform to the equilibrium (p > 0.05). The overall number of effective alleles was close to 2, except for rs638185407 (T > A), rs640683953 (A > C), and rs654358008 (G > C), which had relatively low effective alleles. As a result, the population would exhibit a more even distribution (Table 1).

### 3.3. Linkage disequilibrium analysis and haplotype construction

**Figure 3.**
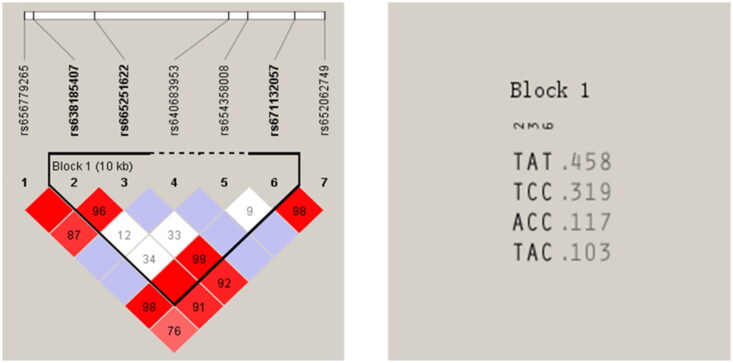
Linkage disequilibrium analysis of *IGF2BP1* gene polymorphic loci, and the corresponding haplotype frequency.

Linkage disequilibrium analysis and haplotype construction were conducted using Haploview 4.2, based on the SNP locations and the typing data (Figure 3). The results show a strong linkage disequilibrium among the rs638185407 (T > A), rs665251622 (A > C) and rs671132057 (T > C) (D’(rs638185407, rs665251622) = 0.962, D’(rs665251622, rs671132057) = 0.991 and D’(rs638185407, rs671132057) = 1.000). In addition, based on these three SNPs, four haplotypes were constructed between the populations. The frequency of the TAT haplotype was the highest at 0.458, followed by the TCC and ACC haplotypes at 0.319 and 0.117 respectively, and the TAC haplotype had the lowest frequency of 0.103.

### 3.4. Associations of SNPs and combination genotypes with growth traits in goats

To investigate whether these seven SNPs related to the growth traits of the Nanjiang Yellow goat, we analyzed the association of SNPs and combination genotypes with growth traits. The genotypes of SNPs in linkage disequilibrium were combined to form combination genotypes. However, to increase the reliability of the data analysis, only samples with all allele frequencies greater than 5% at the corresponding locus (rs656779265(G > A), rs638185407(T > A), rs665251622(A > C), rs671132057(T > C), rs652062749(G > A)) were selected for the association analysis of SNPs with growth traits in Nanjiang Yellow goats (Table 1). Furthermore, the *P-*values of the genotypic effect for the association of selected SNPs and combination genotypes with growth traits were shown in Supplementary Table S5.^20^

#### 3.4.1. Association analysis of five SNPs with growth traits

According to the results of the correlation analysis of growth traits in Nanjiang Yellow goats by month of age (Tables 2 and 3), individuals with the GA genotype at rs656779265 (G > A) showed significantly higher values for BW-2, BL-2, BH-2, CC-6, and CC-18 compared to those with the AA genotype (*P* < 0.05). Furthermore, the value of BW-2 was found to be significantly elevated in individuals with the GA genotype compared to those with the GG genotype (*P* < 0.05). However, the CC-4 value was significantly greater (*P* < 0.05) in individuals with the GG genotype compared to those with the GA genotype, specifically for the rs656779265 (G > A) locus.

**Table 2.** Association analysis of five SNPs with growth traits across different months in the Nanjiang Yellow goat population.

Locus	rs656779265(G > A)			rs638185407(T > A)			rs665251622(A > C)			rs671132057(T > C)			rs652062749(G > A)		
Genotype	AA	GA	GG	AA	AT	TT	AA	CA	CC	CT	CC	TT	AG	GG	AA
Number	51	97	50	3	48	145	56	101	39	94	56	45	77	99	15
BW-0	2.26 ± 0.27^1^	2.26 ± 0.30	2.21 ± 0.32	1.98 ± 0.68	2.30 ± 0.33	2.24 ± 0.28	2.26 ± 0.30	2.24 ± 0.30	2.26 ± 0.30	2.27 ± 0.30	2.22 ± 0.31	2.24 ± 0.29	2.23 ± 0.28	2.27 ± 0.32	2.26 ± 0.23
BW-2	11.11 ± 0.96^b^	11.51 ± 0.94^a^	11.15 ± 0.84^b^	11.00 ± 0.00	11.49 ± 1.03	11.27 ± 0.91	11.21 ± 1.00	11.40 ± 0.90	11.28 ± 0.94	11.46 ± 0.96^a^	11.26 ± 0.88^ab^	11.03 ± 0.90^b^	11.34 ± 0.86	11.31 ± 1.01	11.20 ± 0.98
BL-2	46.94 ± 1.43^b^	47.52 ± 1.68^a^	47.14 ± 1.68^ab^	47.00 ± 1.73	47.50 ± 1.65	47.21 ± 1.64	47.14 ± 1.73	47.37 ± 1.60	47.26 ± 1.60	47.50 ± 1.75^a^	47.30 ± 1.64^ab^	46.78 ± 1.29^b^	47.30 ± 1.48	47.28 ± 1.73	47.00 ± 1.89
BH-2	45.08 ± 1.13^b^	45.58 ± 1.42^a^	45.32 ± 1.35^ab^	44.67 ± 1.53	45.65 ± 1.34	45.31 ± 1.34	45.36 ± 1.52	45.43 ± 1.25	45.36 ± 1.35	45.50 ± 1.45	45.43 ± 1.26	45.02 ± 1.16	45.34 ± 1.21	45.40 ± 1.46	45.40 ± 1.35
CC-2	50.94 ± 1.38	51.34 ± 1.53	51.32 ± 1.59	49.33 ± 1.15^b^	51.61 ± 1.71^a^	51.15 ± 1.41^a^	51.16 ± 1.45	51.18 ± 1.49	51.50 ± 1.64	51.21 ± 1.55	51.46 ± 1.55	50.91 ± 1.36	51.26 ± 1.40	51.18 ± 1.57	51.53 ± 1.51
BW-6	25.75 ± 4.12	26.30 ± 4.21	25.50 ± 4.68	26.67 ± 4.93	26.76 ± 4.58	25.71 ± 4.20	26.01 ± 4.40	26.25 ± 4.16	25.35 ± 4.57	26.30 ± 4.33	25.70 ± 4.45	25.50 ± 4.02	25.62 ± 3.92	26.38 ± 4.51	24.90 ± 4.50
BL-6	59.02 ± 5.45^b^	59.44 ± 5.20	58.66 ± 5.68	58.67 ± 8.14	60.15 ± 5.88	58.58 ± 5.16	59.13 ± 5.40^ab^	59.48 ± 5.34^a^	58.46 ± 5.53^b^	59.51 ± 5.34	58.71 ± 5.48	58.80 ± 5.37	58.74 ± 5.04^b^	59.62 ± 5.68^a^	57.80 ± 5.35^b^
BH-6	56.02 ± 4.78	56.32 ± 4.64	55.94 ± 4.75	56.00 ± 6.24	56.85 ± 5.16	55.96 ± 4.52	56.21 ± 4.84	56.38 ± 4.71	55.59 ± 4.53	56.46 ± 4.74	55.95 ± 4.64	55.71 ± 4.66	55.77 ± 4.20	56.59 ± 5.07	55.27 ± 4.59
CC-6	65.08 ± 4.45	68.38 ± 4.40^a^	65.12 ± 4.68^b^	66.33 ± 6.51^ab^	66.36 ± 4.77^a^	64.88 ± 4.30^b^	65.25 ± 4.53	65.46 ± 4.49	64.87 ± 4.39	65.49 ± 4.59	65.13 ± 4.45	64.82 ± 4.28	64.86 ± 4.30^ab^	65.65 ± 4.66^a^	63.93 ± 3.77^b^
BW-12	34.07 ± 4.86	34.54 ± 4.85	33.72 ± 5.26	34.33 ± 5.69	35.08 ± 5.35	33.96 ± 4.81	34.36 ± 5.20	34.48 ± 4.73	33.62 ± 5.15	34.56 ± 5.05	33.95 ± 4.97	33.77 ± 4.71	33.92 ± 4.34	34.57 ± 5.27	32.90 ± 5.11
BL-12	65.90 ± 5.20	66.27 ± 5.07	66.08 ± 5.38	66.67 ± 5.03^b^	66.96 ± 5.76^a^	56.89 ± 4.97^b^	66.32 ± 5.30	66.16 ± 5.13	66.00 ± 5.18	66.30 ± 5.33	66.09 ± 5.01	65.76 ± 4.95	65.71 ± 4.67^b^	66.62 ± 5.47^a^	64.87 ± 5.28^b^
BH-12	62.78 ± 4.38	63.27 ± 4.33	63.22 ± 4.79	63.33 ± 4.04	63.71 ± 4.96	62.97 ± 4.29	63.25 ± 4.54	63.28 ± 4.41	62.82 ± 4.47	63.31 ± 4.54	63.13 ± 4.40	62.71 ± 4.23	62.83 ± 3.91^ab^	63.51 ± 4.78^a^	62.00 ± 4.71^b^
CC-12	74.76 ± 5.17	75.60 ± 5.07	75.05 ± 53.8	75.33 ± 7.57	76.14 ± 5.72	75.01 ± 4.91	75.23 ± 5.17	75.64 ± 5.16	74.63 ± 5.06	75.58 ± 5.23	75.35 ± 5.22	74.58 ± 5.01	75.00 ± 4.62	75.58 ± 5.50	73.77 ± 5.28
BW-18	47.21 ± 7.07	48.59 ± 7.09	47.48 ± 7.63	47.17 ± 9.00	48.96 ± 7.55	47.70 ± 7.12	47.92 ± 7.80^ab^	48.47 ± 6.98^a^	46.99 ± 7.06^b^	48.55 ± 7.30^a^	47.48 ± 7.01^ab^	46.86 ± 6.84^b^	47.56 ± 6.64^ab^	48.54 ± 7.76^a^	46.10 ± 6.54^b^
BL-18	73.08 ± 5.63	73.79 ± 5.28	72.53 ± 4.92	72.67 ± 5.03	74.50 ± 5.79	72.97 ± 5.10	72.98 ± 5.22	73.70 ± 5.37	72.79 ± 5.29	73.82 ± 5.46	73.20 ± 5.38	72.27 ± 4.64	72.99 ± 4.96^ab^	73.69 ± 5.47^a^	71.80 ± 4.99^b^
BH-18	68.75 ± 3.69	69.60 ± 4.26	69.14 ± 4.59	69.00 ± 6.08	70.10 ± 4.63	69.03 ± 4.02	69.18 ± 4.05	69.45 ± 4.18	69.13 ± 4.56	69.63 ± 4.36	69.16 ± 4.43	68.53 ± 3.40	69.01 ± 3.79	69.51 ± 4.35	68.67 ± 4.88
CC-18	85.03 ± 4.88^b^	86.30 ± 4.63^a^	85.11 ± 5.40^ab^	83.33 ± 7.57^ab^	87.01 ± 5.10^a^	85.32 ± 4.74^b^	85.43 ± 5.06^b^	86.17 ± 4.69^a^	84.97 ± 5.22^b^	86.30 ± 4.82^a^	85.24 ± 4.88^b^	84.74 ± 4.79^b^	85.51 ± 4.59^ab^	85.94 ± 5.13^a^	84.20 ± 4.55^b^

BW-NUMBER (kg) = body weight for different months (kg); BL-NUMBER (cm) = body length for different months (cm); BH-NUMBER (cm) = body height for different months (cm); CC-NUMBER (cm) = chest circumference for different months (cm).

^1^ explains values are exhibited as means ± standard deviation.

Different lowercase letters in the same column indicate significant differences (*P* < 0.05), and no letter indicates no significant difference (*P* > 0.05).

**Table 3.** Association analysis of five SNPs with growth traits in the Nanjiang Yellow goat population at four months of age.

locus	Genotype	Number	BW-4	BL-4	BH-4	CC-4
rs656779265 (G > A)	AA	73	14.33 ± 2.11^1^	51.63 ± 2.53	50.36 ± 2.85	54.51 ± 3.02^ab^
	GA	24	13.98 ± 2.06	51.48 ± 2.83	50.04 ± 2.88	54.20 ± 2.99^b^
	GG	32	14.88 ± 2.38	52.59 ± 2.74	50.96 ± 2.53	55.73 ± 2.86^a^
rs638185407 (T > A)	AA	1	18.80 ± 0.00	54.50 ± 0.00	53.50 ± 0.00^ab^	60.00 ± 0.00
	AT	21	13.43 ± 2.01	50.57 ± 2.77	48.92 ± 2.86^b^	53.80 ± 3.09
	TT	105	14.41 ± 2.15	51.98 ± 2.74	50.59 ± 2.73^a^	54.71 ± 2.96
rs665251622 (A > C)	AA	76	14.42 ± 2.42^b^	51.62 ± 2.7	50.81 ± 2.97^ab^	54.75 ± 3.49
	CA	14	13.99 ± 1.93^b^	51.57 ± 2.82	49.86 ± 2.73^b^	54.31 ± 2.67
	CC	38	15.44 ± 2.39^a^	53.21 ± 2.47	51.64 ± 2.20^a^	55.84 ± 3.19
rs671132057 (T > C)	CC	68	14.63 ± 2.38	52.52 ± 2.92	50.68 ± 2.50	55.59 ± 2.82^a^
	CT	37	14.09 ± 2.10	51.5 ± 2.79	50.05 ± 2.90	54.19 ± 3.04^b^
	TT	21	14.30 ± 2.15	51.46 ± 2.49	50.65 ± 3.08	54.40 ± 3.15^ab^
rs652062749 (G > A)	AA	49	15.10 ± 2.26	54.26 ± 1.78^a^	51.00 ± 1.93	54.46 ± 2.89
	AG	67	14.41 ± 1.94	52.02 ± 2.68^b^	50.53 ± 2.76	54.88 ± 2.78
	GG	7	13.96 ± 2.26	51.18 ± 2.78^b^	49.99 ± 2.92	54.38 ± 3.16

BW-4 (kg) = body weight at four months of age (kg); BL-4 (cm) = body length at four months of age (cm); BH-4 (cm) = body height at four months of age (cm); CC-4 (cm) = chest circumference at four months of age (cm).

^1^explaining values are exhibited as means ± standard deviation.

Different lowercase letters in the same column indicate significant differences (*P* < 0.05), and no letter indicates no significant difference (*P* > 0.05).

Individuals with TT genotype at rs638185407 (T > A) displayed significantly higher CC-2 values than AA genotype (*P* < 0.05). Given that the sample size for the AA genotype at rs638185407 (T > A) at four months of age was less than three, the post-hoc test for it was not conducted. Therefore, individuals with the TT genotype at rs638185407 (T > A) had significantly higher BH-4 value compared to those with the AT genotype (*P* < 0.05). Additionally, individuals with the AT genotype at rs638185407 (T > A) exhibited significantly higher CC-2 and BL-12 values than those with the AA genotype (*P* < 0.05). Furthermore, individuals with the AT genotype at rs638185407 (T > A) exhibited significantly higher CC-6, BL-12, and CC-18 values than those with the TT genotype (*P* < 0.05).

For the rs665251622 (A > C), individuals with the CA genotype showed significantly higher BL-6, BW-18, and CC-18 values than those with the CC genotype (*P* < 0.05). Nevertheless, individuals with the CC genotype at rs665251622 (A > C) exhibited significantly higher BW-4, BL-4, and BH-4 values than those with the CA genotype (*P* < 0.05), and these advantages were not sustained over time. Additionally, they also demonstrated significantly higher BW-4 and BL-4 values than those with the AA genotype (*P* < 0.05). Moreover, individuals with the CA genotype at rs665251622 (A > C) had significantly higher CC-18 values compared to those with the AA genotype (*P* < 0.05).

Individuals with the CT genotype at rs671132057 (T > C) demonstrated significantly greater values for BW-2, BL-2, BL-18, and CC-18 than those with the TT genotype (*P* < 0.05). Additionally, the CC-4 value was found to be significantly inferior, whereas the CC-18 value was significantly greater in individuals with the CT genotype compared to those with the CC genotype *(P < 0.05*).

At rs652062749 (G > A), individuals with the AA genotype displayed a significantly higher BL-4 value than both the GG (*P* < 0.05) and AG genotypes (*P* < 0.05), although this advantage was not sustained over time. Conversely, individuals with the GG genotype individuals at rs652062749 (G > A) had significantly greater values for BL-6, CC-6, BL-12, BH-12, BW-18, BL-18, and CC-18 compared to the AA genotype (*P* < 0.05). Furthermore, the BL-6 and BL-12 values were also significantly elevated in GG individuals compared to AG genotypes (*P* < 0.05).

#### 3.4.2. Association analysis of combination genotypes with growth traits

Combination genotypes are formed by combining genotypes of three SNPs (rs638185407 (T > A), rs665251622 (A > C) and rs671132057 (T > C)) in linkage disequilibrium. To ensure accurate association analysis, only combination genotypes with a sample size exceeding three individuals were selected to evaluate their relationship with the growth traits of Nanjiang Yellow goats.

The analysis of birth weight across combination genotypes revealed that individuals with the ATCACT genotype exhibited the highest average birth weight. The birth weight was significantly higher than that of the TTCACT, TTAATT, and TTCACC genotypes (*P* < 0.05) (Table 4).

**Table 4.** Association analysis of combination genotypes and birth weight of Nanjiang Yellow goat.

Combination genotypes	Birth weight (kg)
ATCACT (44)	2.35 ± 0.28^a^
TTAACT (21)	2.32 ± 0.35^ab^
TTAATT (66)	2.24 ± 0.29^b^
TTCCCC (33)	2.24 ± 0.26^ab^
TTCACC (27)	2.23 ± 0.20^b^
TTCACT (93)	2.22 ± 0.29^b^
TTAACC (4)	2.20 ± 0.14^ab^
ATCACC (10)	2.08 ± 0.58^ab^
AACCCC (4)	1.98 ± 0.68^ab^

Different lowercase letters in the same column indicate significant differences (*P* < 0.05), and no letter indicates no significant difference (*P* > 0.05).

The analysis of the association between combination genotypes and growth traits at two months of age revealed that the goats with the TTAACT combination genotype exhibited the greatest average values across all parameters. The body weight of the ATCACT, TTAACT, and TTCACT genotypes were significantly higher than that of the TTAATT genotype (*P* < 0.05). The body length and body height of the TTAACT were found to be significantly higher than those of the TTAATT, TTCACT, and TTCCCC genotypes (*P* < 0.05). Furthermore, the body length and body height of the ATCACT genotype were significantly higher than those of the TTAATT genotype (*P* < 0.05) (Table 5).

**Table 5.** Association analysis of combination genotypes and growth traits of Nanjiang Yellow goat at two months of age.

Combination genotypes	Two months of age			
	Body weight (kg)	Body length (cm)	Body height (cm)	Chest circumference (cm)
ATCACC (5)	11.20 ± 1.10^ab^	47.20 ± 2.39^abc^	45.40 ± 1.34^abc^	51.40 ± 1.67
ATCACT (31)	11.52 ± 1.08^a^	47.48 ± 1.61^c^	45.65 ± 1.40^c^	51.40 ± 1.72
TTAACT (8)	11.88 ± 1.25^a^	48.88 ± 2.70^ac^	46.88 ± 2.42^ac^	52.25 ± 1.28
TTAATT (45)	11.03 ± 0.90^b^	46.78 ± 1.29^b^	45.02 ± 1.16^b^	50.91 ± 1.36
TTCACC (10)	11.10 ± 0.52^ab^	47.50 ± 1.51^abc^	45.60 ± 1.07^abc^	51.25 ± 1.03
TTCACT (53)	11.40 ± 0.84^a^	47.28 ± 1.61^bc^	45.23 ± 1.19^bc^	51.02 ± 1.39
TTCCCC (23)	11.13 ± 0.96^ab^	47.00 ± 1.62^bc^	45.17 ± 1.34^bc^	51.35 ± 1.47

Different lowercase letters in the same column indicate significant differences (*P* < 0.05), and no letter indicates no significant difference (*P* > 0.05).

At four months of age, individuals with the TTCCCC combination genotype demonstrated significantly greater body length value than those with the ATCACT, TTCACC, TTAATT, TTAACT, and TTCACT genotypes (*P* < 0.05). Additionally, individuals with the TTCCCC combination genotype exhibited significantly greater body height than those with the ATCACC, ATCACT, and TTCACC genotypes (*P* < 0.05). Meanwhile, their chest circumference was larger than that of individuals with the ATCACT genotype (*P* < 0.05). Furthermore, the ATCACT genotype exhibited significantly reduced body height in comparison to other genotypes, with the exception of the TTAACT genotype (*P* < 0.05) (Table 6).

**Table 6.** Association analysis of combination genotypes and growth traits of Nanjiang Yellow goat at four months of age.

Combination genotypes	Four months of age			
	Body weight (kg)	Body length (cm)	Body height (cm)	Chest circumference (cm)
ATCACC (5)	14.24 ± 1.71	52.76 ± 1.97^abc^	49.52 ± 1.94^b^	55.96 ± 2.63
ATCACT (13)	13.05 ± 2.24	49.83 ± 2.86^c^	48.37 ± 3.21^c^	52.82 ± 3.13
TTAACT (13)	14.24 ± 2.68	51.27 ± 2.55^bc^	50.32 ± 2.61^abc^	54.63 ± 4.04
TTAATT (21)	14.30 ± 2.15	51.46 ± 2.49^bc^	50.65 ± 3.08^ab^	54.40 ± 3.15
TTCACC (17)	13.81 ± 1.91	51.28 ± 2.83^bc^	49.90 ± 2.06^b^	54.74 ± 2.12
TTCACT (40)	14.37 ± 1.83	52.13 ± 2.74^b^	50.43 ± 2.80^ab^	54.43 ± 2.63
TTCCCC (10)	15.70 ± 2.34	54.03 ± 1.92^a^	52.15 ± 2.09^a^	55.96 ± 3.34

Different lowercase letters in the same column indicate significant differences (*P* < 0.05), and no letter indicates no significant difference (*P* > 0.05).

A correlation between combination genotypes and growth traits at the age of six months revealed that the values of the ATCACT combination genotype were significantly superior to the TTCCCC combination genotype in terms of body weight and body length (*P* < 0.05). Furthermore, the body height of individuals with the TTAACT genotype was also significantly higher than that with the TTCCCC genotype (*P* < 0.05). Additionally, the ATCACT combination genotype exhibited a significantly greater value for chest circumference than the TTCACT and TTCCCC genotypes (*P* < 0.05) (Table 7).

**Table 7.** Association analysis of combination genotypes and growth traits of Nanjiang Yellow goat at six months of age.

Combination genotypes	Six months of age			
	Body weight (kg)	Body length (cm)	Body height (cm)	Chest circumference (cm)
ATCACC (5)	26.40 ± 5.81^ab^	59.20 ± 7.36^ab^	57.20 ± 7.05^ab^	65.40 ± 5.13^ab^
ATCACT (31)	27.29 ± 4.31^a^	60.71 ± 5.75^a^	57.19 ± 5.03^ab^	66.82 ± 4.75^a^
TTAACT (8)	28.19 ± 5.74^ab^	60.75 ± 5.65^ab^	58.50 ± 5.63^a^	67.00 ± 5.01^ab^
TTAATT (45)	25.50 ± 4.02^ab^	58.80 ± 5.37^ab^	55.71 ± 4.66^ab^	64.82 ± 4.28^ab^
TTCACC (10)	26.70 ± 3.43^ab^	59.90 ± 4.98^ab^	56.90 ± 4.41^ab^	66.00 ± 4.22^ab^
TTCACT (53)	25.47 ± 4.06^ab^	58.68 ± 5.08^ab^	55.75 ± 4.45^ab^	64.56 ± 4.32^b^
TTCCCC (23)	24.78 ± 4.39^b^	57.78 ± 5.08^b^	55.09 ± 4.06^b^	64.04 ± 3.78^b^

Different lowercase letters in the same column indicate significant differences (*P* < 0.05), and no letter indicates no significant difference (*P* > 0.05).

Correlating the combination genotypes with growth traits at one year of age revealed that individuals with the TTAACT genotype exhibited the highest average values across all growth traits. The TTAACT genotype also demonstrated significantly greater body weight, length, and height than the TTCCCC and TTCACT genotypes (*P* < 0.05). Furthermore, the body weight value was also found to be significantly greater for the ATCACT genotype than for the TTCCCC genotype (*P* < 0.05). Chest circumferences were significantly greater for the value of TTAACT genotype compared to the TTAATT and TTCCCC genotypes(*P* < 0.05) (Table 8).

**Table 8.** Association analysis of combination genotypes and growth traits of Nanjiang Yellow goat at one year of age.

Combination genotypes	One year of age			
	Body weight (kg)	Body length (cm)	Body height (cm)	Chest circumference (cm)
ATCACC (5)	35.30 ± 4.02^abc^	66.80 ± 5.76^ab^	64.00 ± 5.43^ab^	76.40 ± 6.58^ab^
ATCACT (31)	35.53 ± 5.29^ab^	67.06 ± 5.82^ab^	63.87 ± 4.99^ab^	74.83 ± 4.79^ab^
TTAACT (8)	37.31 ± 6.85^a^	69.13 ± 6.51^a^	65.63 ± 5.4^a^	78.13 ± 5.33^a^
TTAATT (45)	33.77 ± 4.71^abc^	65.76 ± 4.95^ab^	62.71 ± 4.23^ab^	74.58 ± 5.01^b^
TTCACC (10)	34.40 ± 5.81^abc^	66.70 ± 5.01^ab^	64.20 ± 4.26^ab^	76.90 ± 4.98^ab^
TTCACT (53)	33.67 ± 4.48^b^	65.51 ± 4.8^b^	62.68 ± 4.11^b^	76.44 ± 5.77^ab^
TTCCCC (23)	32.89 ± 4.85^bc^	65.09 ± 4.44^b^	62.17 ± 3.95^b^	74.24 ± 4.72^b^

Different lowercase letters in the same column indicate significant differences (*P* < 0.05), and no letter indicates no significant difference (*P* > 0.05).

The association of combination genotypes with growth traits at 1.5 years of age revealed that the value of the TTAACT genotype still exhibited the highest average values across all growth traits. The value of the TTAACT genotype exhibited significantly greater body weight than the TTAATT, TTCACT, and TTCCCC genotypes (*P* < 0.05). With regard to body length, the value of the TTAACT genotype was significantly greater than that of the TTAATT and TTCCCC genotypes (*P* < 0.05). Furthermore, the value of the TTAACT genotype for chest circumference was found to be significantly higher than that of the TTCCCC genotype. Additionally, the value of the ATCACT genotype for chest circumference were observed to be significantly greater than that of the TTAATT, TTCACT, and TTCCCC genotypes (*P* < 0.05) (Table 9).

**Table 9.** Association analysis of combination genotypes and growth traits of Nanjiang Yellow goat at 1.5 years of age.

Combination genotypes	1.5 years of age			
	Body Weight(kg)	Body Length(cm)	Body Height(cm)	chest circumference(cm)
ATCACC (5)	49.80 ± 9.42^ab^	75.00 ± 7.31^ab^	70.00 ± 6.16	86.20 ± 6.61^abc^
ATCACT (31)	49.31 ± 7.63^ab^	74.58 ± 5.67^ab^	70.23 ± 4.63	87.58 ± 4.80^ac^
TTAACT (8)	53.00 ± 10.76^a^	76.50 ± 7.11^a^	72.50 ± 5.61	88.50 ± 5.71^a^
TTAATT (45)	46.86 ± 6.84^b^	72.27 ± 4.64^b^	68.53 ± 3.40	84.74 ± 4.79^ab^
TTCACC (10)	49.15 ± 7.2^ab^	74.40 ± 5.62^ab^	69.40 ± 3.84	86.20 ± 3.74^abc^
TTCACT (53)	47.58 ± 6.41^b^	73.08 ± 5.06^ab^	68.98 ± 3.87	85.25 ± 4.57^ab^
TTCCCC (23)	45.80 ± 6.08^b^	71.74 ± 4.71^b^	68.43 ± 4.40	84.24 ± 4.47^b^

Different lowercase letters in the same column indicate significant differences (*P* < 0.05), and no letter indicates no significant difference (*P* > 0.05).

### 3.5. Testing and functional verification of non-coding SNPs in IGF2BP1

**Figure 4.**
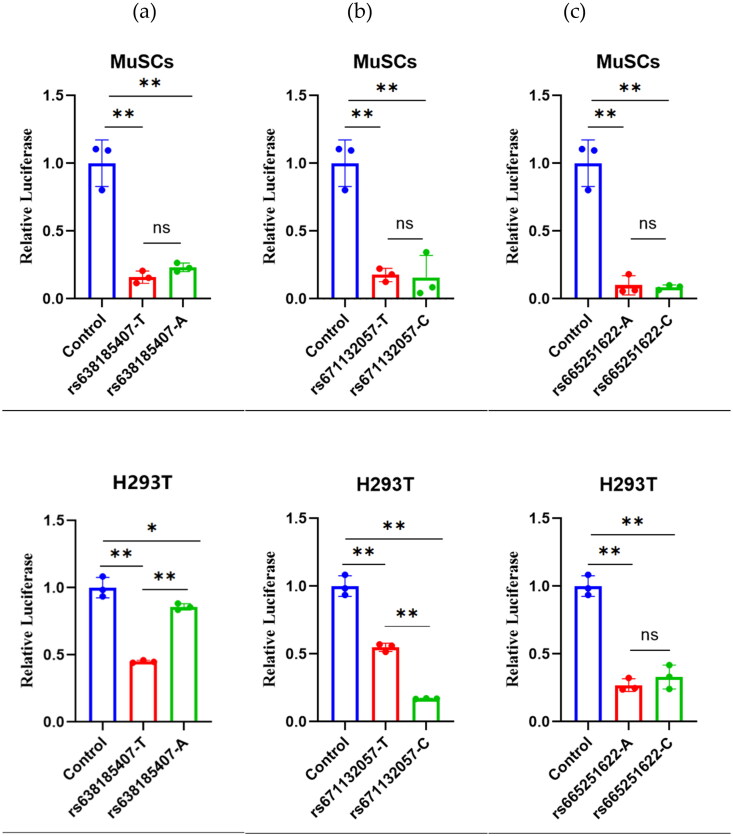
The results of the dual-luciferase assays for three SNPs in two cells, where the red and green represent wild type and mutant type, respectively. Besides, the control is an empty PGL4.23 vector.

Non-coding SNPs have the potential to indirectly regulate the gene expression process, thereby influencing animal phenotype or reproductive performance.^21^^,^^22^ The dual luciferase reporter vector assay is an accurate and reliable method to validate non-coding SNPs in research.^23^^,^^24^ For this study, we performed dual luciferase assays for three strong linkage SNPs in two cells, MuSCs and H293T^25^ (Figure 4). For rs638185407(T > A), the dual luciferase activity of both genotypes in MuSCs cells was highly significantly lower than that of the control (P < 0.01), indicating an inhibitory effect. However, the comparison of the two types was not statistically significant. There was a trend toward elevated activity in the mutant type (A), with the dual-luciferase activity of the wild type being highly significantly lower than that of the control (*P* < 0.01) and the mutant type being significantly lower than the H293T cells (*P* < 0.05). Both the wild and mutant types showed inhibitory effects, with the wild type demonstrating highly significantly lower activity compared to the mutant type (*P* < 0.01).

In terms of rs671132057(T > C), in MuSCs cells, the dual-luciferase activities of both types were highly significantly lower than that of the control (*P* < 0.01), indicating an inhibitory effect. However, the comparison between the two types was not significant, while the mutant type(C) tended to decrease. In H293T cells, both types’ dual-luciferase activities were also highly significantly lower than the control (*P* < 0.01), indicating an inhibitory effect. Furthermore, the mutant activity was highly significantly lower than the wild type (*P* < 0.01).

For rs665251622(A > C), both genotypes showed a highly significant decrease (*P* < 0.01) in dual-luciferase activity within MuSCs cells compared to controls, indicating inhibition. However, there was no significant difference observed between the two genotypes. In contrast, the wild-type genotype exhibited a highly significant reduction (*P* < 0.01) in dual-luciferase activity in H293T cells compared to controls, also indicating inhibition. Similar to the MuSCs cells, there was no significant difference between the two genotypes, although there was a trend suggesting increased activity in the mutant type (the C type).

## 4. Discussion

*IGF2BP1* is a crucial candidate gene affecting animal growth and development. Consequently, association analysis studies of its polymorphisms with growth traits have also been conducted in numerous livestock and poultry species. Several studies have demonstrated a direct correlation between the degree of genetic heterozygosity and the population’s genetic diversity. This implies that the higher the degree of heterozygosity, the richer the genetic diversity and the greater the selection pressure.^26–28^ In this experiment, a total of three SNPs, rs638185407(T > A), rs640683953(A > C), and rs654358008(G > C), exhibited a low degree of polymorphism (0.25 ≥ PIC), which indicated that the enrichment of genetic diversity was low at these three SNPs.^29^^,^^30^ Except for the three SNPs mentioned above, the effective numbers of alleles for the remaining SNPs were close to 2, indicating that these SNPs exhibited a high degree of variation and richness of genetic diversity.^31^ The Hardy-Weinberg equilibrium test revealed all SNPs were in equilibrium, indicating that these SNPs had not been subjected to high-intensity artificial selection.^32^ This was because the experimental samples were all harvested from the same region, with a concentrated growth environment and a long period of closed breeding.

Many researches have demonstrated that the majority of SNPs do not directly influence protein sequences. Instead, they primarily impact individual organisms through mutations, resulting in the elimination of various transcription factor binding sites and the emergence of new binding sites. These alterations enable transcription factors to function, thereby influencing the regulation of gene expression.^33–35^ In this study, six SNPs (rs638185407(T > A), rs665251622(A > C), rs640683953(A > C), rs654358008(G > C), rs671132057(T > C), and rs652062749(G > A)) were found to have altered transcription factor binding sites after mutation (Supplementary Table S4), presumably affecting the expression of *IGF2BP1* gene in Nanjiang Yellow goat. The correlation between the pre-mutation and post-mutation changes of transcription factors at the three strongly interlinked SNP sites and the results of the dual luciferase assay were investigated, as well as their correlation with the results of the growth trait association analysis.

MEF2 plays various roles in muscle cells.^36–38^ It has been found to act not only in studies of Drosophila muscle by collaborating with PAR structural domain protein 1,^39^ but also to affect Ashtan yak growth and development through MEF2A gene copy number variation.^40^ In this study, three new Myocyte enhancer factor-2(MEF2) family transcription factors, MEF2C, MEF2B, and MEF2D, were identified after mutation at rs665251622(A > C). Preliminary validation using MuSCs and H293T cells revealed that the difference in dual-luciferase activities between the mutant type and wild type at rs665251622(A > C) was not significant, which is consistent with the previous association analysis results performed in agreement. This indicates that this locus is not a potential locus to affect the growth traits of Nanjiang Yellow goats.

The transcription factor JUNB, as a member of the dimeric transcription factor AP-1 (Activator Protein-1) family, inhibits cell proliferation, induces cellular senescence, and suppresses tumor development.^41–44^ The prediction of transcription factor binding sites revealed that the wild type of rs638185407(T > A) has a JUNB binding site, whereas the mutant type has lost the JUNB binding site. Losing the JUNB binding site may lead to the elevation of the dual-luciferase of rs638185407(T > A) in the mutant type compared to the wild type. However, the data obtained from the association analysis indicated heterozygous dominance, which was inconsistent with the results of the dual-luciferase activity assay. This indicates that this locus may affect the growth traits of Nanjiang Yellow goats through other pathways, and the specific reasons need to be further investigated.

Furthermore, the POU family of structural domain factors is an essential class of regulators with specific structural domains and POU-HDs, which were necessary for high-affinity DNA sequence recognition.^45^ Their main functions are to inhibit or promote cell proliferation, migration, and differentiation.^46–48^ The POU family genes, including POU1F1, POU2F1, and POU3F1, were identified before the rs671132057(T > C) mutation. However, the POU family of genes disappeared from this SNP after the mutation, which may be responsible for the significant decrease in dual luciferase activity compared to before the mutation. As for the association analysis results, individuals with this locus showed heterozygous dominance in all growth traits. The results of cellular-level validation and individual-level analysis were inconsistent, indicating that the effect of the mutation locus on the phenotype of an individual is a complex process. Therefore, the specific mechanism by which this locus affects the growth traits of the Nanjiang Yellow goats remains to be further investigated.

## Supplementary Material

R3 Supplementary Material File.docx

## Data Availability

The data from this study are exhibited in this manuscript and Supplementary.

## Supplementary material

The following supporting information can be found in manuscript file named Supplementary Material File, Figure S1: Schematic diagram of PGL 4.23 vector recombinant plasmid: (a): PGL 4.23 vector plasmid containing rs671132057 (T > C) (b): PGL 4.23 vector plasmid containing the rs638185407 (T > A) (c): PGL 4.23 vector plasmid containing the rs665251622 (A > C); Figure S2: Gel electrophoresis of genomic DNA samples; Table S1: Enzyme-cut primer information; Table S2: Primer information for amplification of 7 SNPs on the second intron of *IGF2BP1*; Table S3: Primers used for Mass-array genotyping; Table S4: Prediction of binding sites for transcription factors; Table S5: All *P-*values of genotypic effects from Table 2 to Table 9.
