# Supplementary material for: Identification of SNPs in the second intron of IGF2BP1 and their Association with growth traits in Nanjiang Yellow goat
Source: Anim Biotechnol. 2025 Feb 17;36(1):2461176. doi: 10.1080/10495398.2025.2461176 (PMC12674248; doi:10.1080/10495398.2025.2461176)

**Supplementary Table S1:** Enzyme-cut primer information

| **locus** | **Primer sequence(5'–3')** | **Tm（℃）** | **product length（bp）** |
| --- | --- | --- | --- |
| rs671132057（T>C） | ggGGTACCgcctgcaaatactcccactc | 62.4 | 488 |
|  | cccAAGCTTcctcctgtcaactggtgtcat |  |  |
| rs638185407（T>A） | cggGGTACCgggcacaggatttaacttgc | 59.8 | 480 |
|  | cccAAGCTTttcacctcgcacttcatctg |  |  |
| rs665251622（A>C） | cggGGTACCgggtgtcatcagttaaagat | 58 | 352 |
|  | cccAAGCTTgttacaagcctacagaatca |  |  |

**Note:** The primer sequences are in capital letters, and the protective bases and common sequences are in lowercase letters.

**Supplementary Table S2:** Primer information for amplification of 7 SNPs on the second intron of IGF2BP1

| **locus** | **Primer sequence（5' – 3'）** | **Tm（℃）** | **product length（bp）** |
| --- | --- | --- | --- |
| rs656779265（G>A） | F:AGCAATAGCAAGTTCCAGTT | 60.4 | 404 |
|  | R:CAATCATCCAGCCGTATTATAG |  |  |
| rs638185407（T>A） | F:GGGCACAGGATTTAACTTGC | 59.8 | 480 |
|  | R:TTCACCTCGCACTTCATCTG |  |  |
| rs665251622（A>C） | F:GGGTGTCATCAGTTAAAGAT | 58.0 | 352 |
|  | R:GTTACAAGCCTACAGAATCA |  |  |
| rs640683953（A>C） | F:TGTTCAAGCCTCTTCTTCC | 62 | 370 |
|  | R:CACCTGTGTCTAGTCCATAC |  |  |
| rs654358008（G>C） | F:CCTCTGCCTCTTCACTCCAC | 58.0 | 460 |
|  | R:TGCCTTCTCTTCGCTTTTTC |  |  |
| rs671132057（T>C） | F:GCCTGCAAATACTCCCACTC | 56.0 | 555 |
|  | R:CCTCCTGTCAACTGGTGTCAT |  |  |
| rs652062749（G>A） | F:TAGGAACTAGGTTGGAAGGT | 63.0 | 546 |
|  | R:GTTTGTGAGGACTGGGTAC |  |  |

**Supplementary Table S3:** Primers used for Mass-array genotyping

| **locus** | **forward primer** | **reverse primer** | **extension primer** |
| --- | --- | --- | --- |
| rs656779265 | ACGTTGGATGGGCTATACAAGCTTCTGCTG | ACGTTGGATGCTGGTTTCTGCTCATCTCAC | ggtCTCATCTCACCAAACTTC |
| rs638185407 | ACGTTGGATGAGTGGGCTCTCTAGTCAAAC | ACGTTGGATGTTGGAGAGCAAGCAAAGGAG | AGGAATAAATCTTTTGCTCAATT |
| rs665251622 | ACGTTGGATGCTGAGCCTGTTTTTGAGCTG | ACGTTGGATGACAAGAGGTTGATTTGAGCC | tcccCTAAACCACAGAATATTGAGA |
| rs640683953 | ACGTTGGATGAAAGCTGACTCTGCTTGTTC | ACGTTGGATGACCACCTGGAGTTTGTTCAC | cACTTTTTAGCAGGTCCAA |
| rs654358008 | ACGTTGGATGTAGAAGGTCTGCACCATGGG | ACGTTGGATGAGCCCCCAGATCCCAAACCT | ttaaCCGGCCTTCCGCACG |
| rs671132057 | ACGTTGGATGGATTTATGGAGACGCTGAAG | ACGTTGGATGAGGCAAACCCATTCCTGAAG | GGCAAAATTTTTGTCTGTTTTGAATA |
| rs652062749 | ACGTTGGATGTTGTGGATGTATAGGACAGG | ACGTTGGATGGCACCATTGTTCATTTTGCT | ccTTTTGCTTAAAATACATTAGTCATA |

**Supplementary Table S4:** Prediction of binding sites for transcription factors

| **locus** | **pre-mutation transcription binding factor(Score>8.5)** | **post-mutation transcription binding factor(Score>8.5)** |
| --- | --- | --- |
| rs656779265（G>A） | / | / |
| rs638185407（T>A） | BATF::JUN，CEBPD，CEBPA，BATF3，BATF，FOSB::JUNB，FOSL1::JUND，FOSL2::JUNB，Atf3，FOSL2::JUND，DUXA，JUNB，FOS::JUN | CEBPD，ISX，RAX，HESX1，TLX2，Shox2，MSX1，EN2，MSX2，Alx1，CEBPA，ESX1，ZNF652， |
| rs665251622（A>C） | Arid5a，MEF2A，Foxj3，OLIG3 | MEF2A，MEF2C，MEF2B，Arid5a，Foxj3，MEF2D |
| rs640683953（A>C） | JUN::JUNB，NR1D2，NFYB，FOS::JUN，NR2C1，FOSL1::JUND，JDP2，NFYB，FOSL1::JUN，FOSL2，NFE2，Jun，JUND | NR2C1，RXRA::VDR，NR2C2，NR1I2，INSM1，Sox2，NR2F1 |
| rs654358008（G>C） | Nrf1，Mycn，Ahr::Arnt，MYC，ARNT::HIF1A | MZF1 |
| rs671132057（T>C） | LIN54，POU2F3，POU5F1B，POU1F1，POU2F1，POU5F1，POU3F1 | LIN54，FOXH1，POU5F1B，POU2F3，MSANTD3，NR3C1，NKX2-2 |
| rs652062749（G>A） | LMX1B，Alx4，Hoxb5，FOSB::JUNB，mix-a，FOSL2::JUNB，Alx1，Lhx3，FOS::JUN，JUNB，BATF，Atf3，MEIS1，BNC2，TFCP2 | Lhx3，PAX7，PAX3，Hoxd8，Alx4，Alx1，mix-a，HNF1A，FOS::JUN，BATF，Atf3 |

**Supplementary Table S5:** All *P-*values of genotypic effects from Table 2 to Table 9

(**a**)

(**b**)

**Supplementary Figure S1:** Schematic diagram of PGL 4.23 vector recombinant plasmid: (**a**): PGL 4.23 vector plasmid containing rs671132057 (T>C). (**b**): PGL 4.23 vector plasmid containing the rs638185407 (T>A). (**c**): PGL 4.23 vector plasmid containing the rs665251622 (A>C).

(**a**)


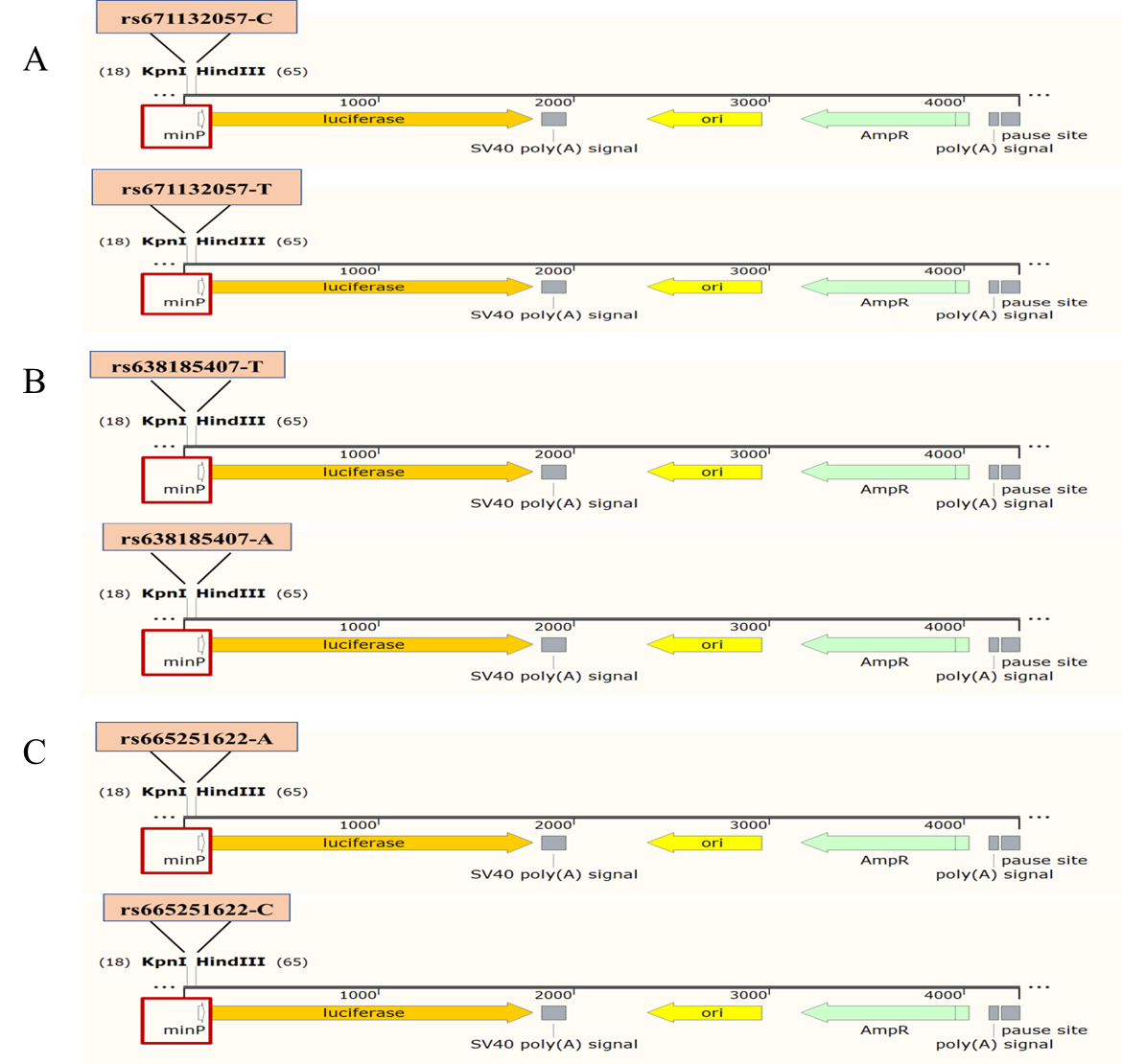


(**b**)


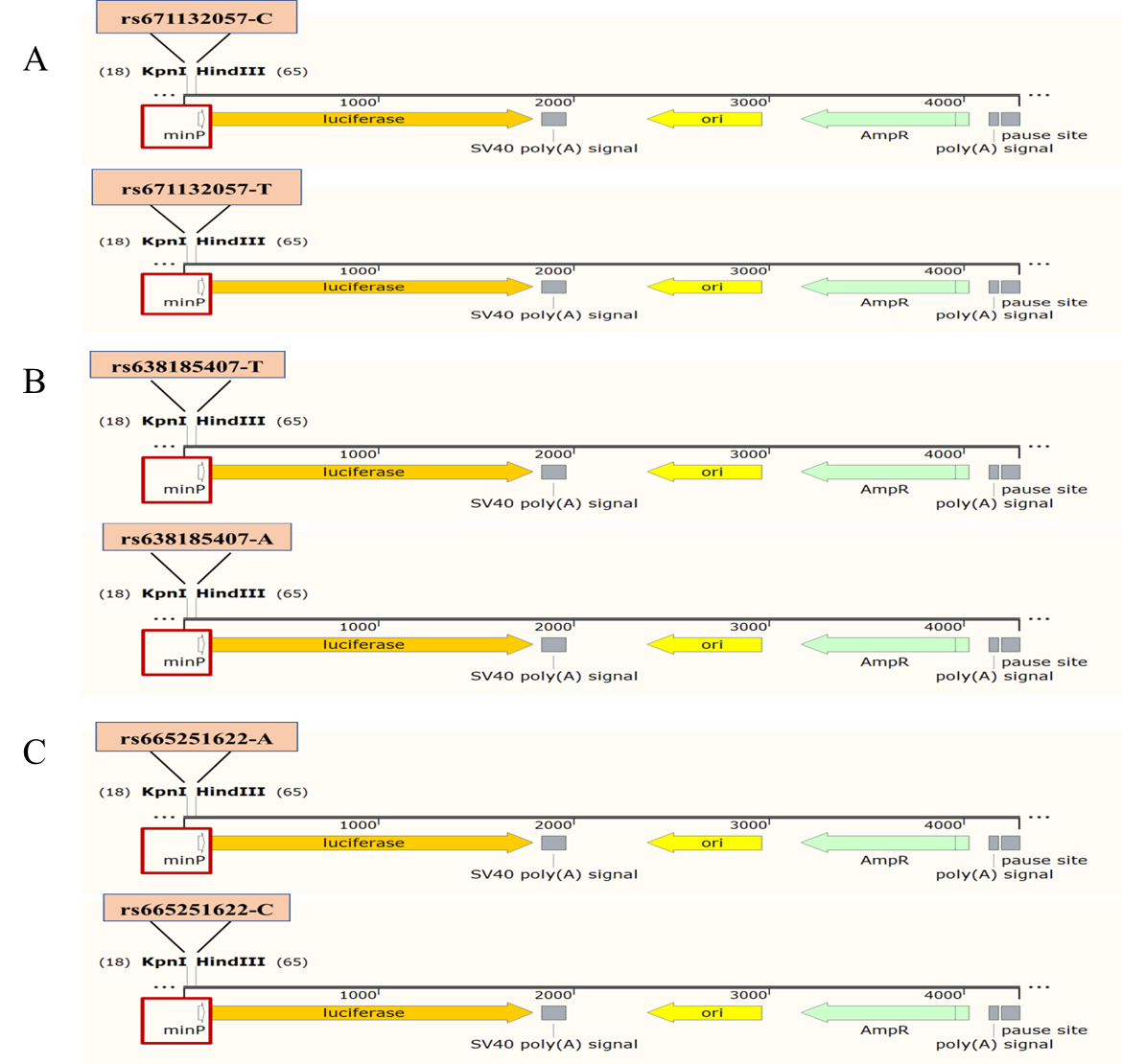


(**c**)


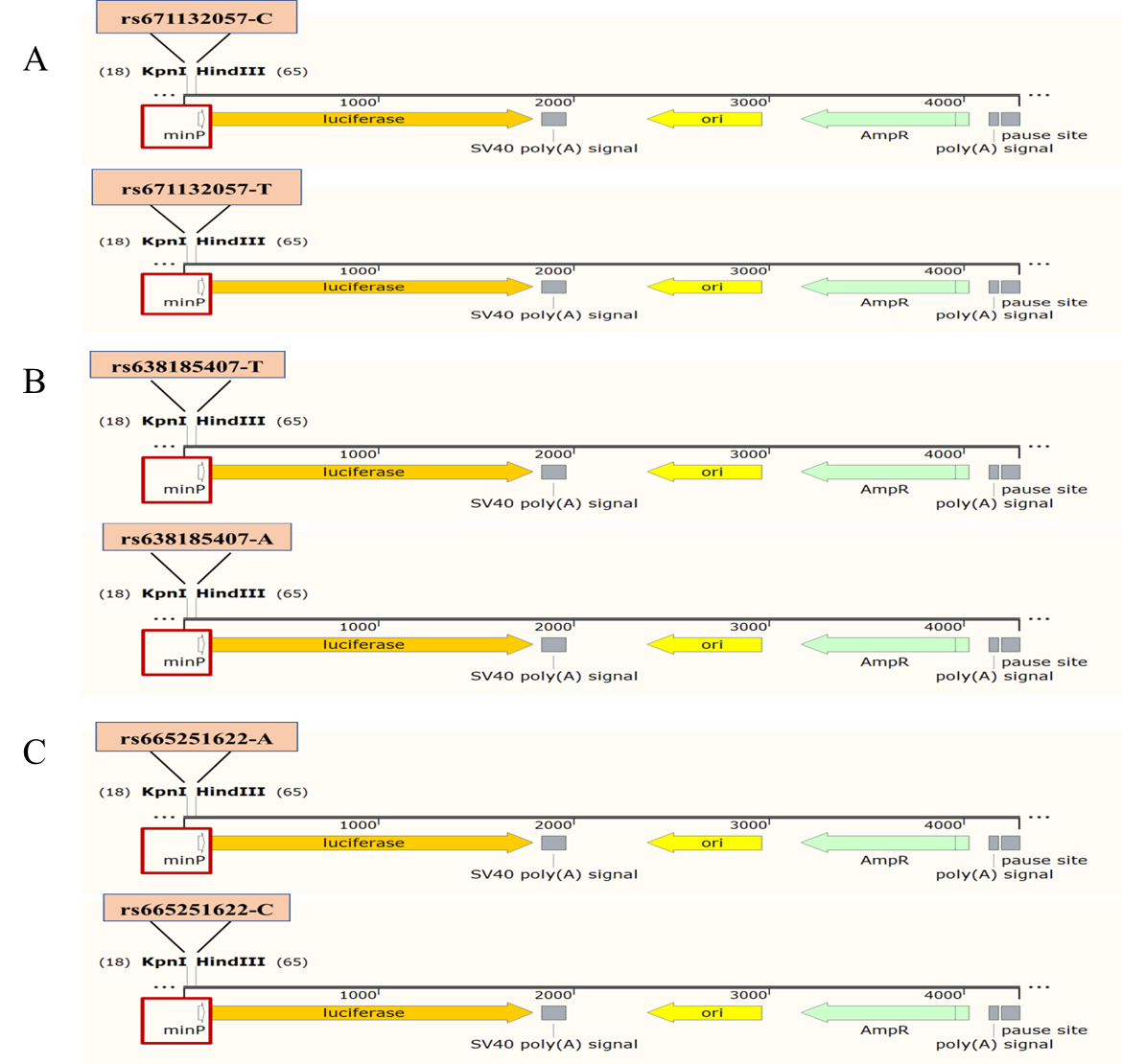


**Supplementary Figure S2:** Gel electrophoresis of genomic DNA samples


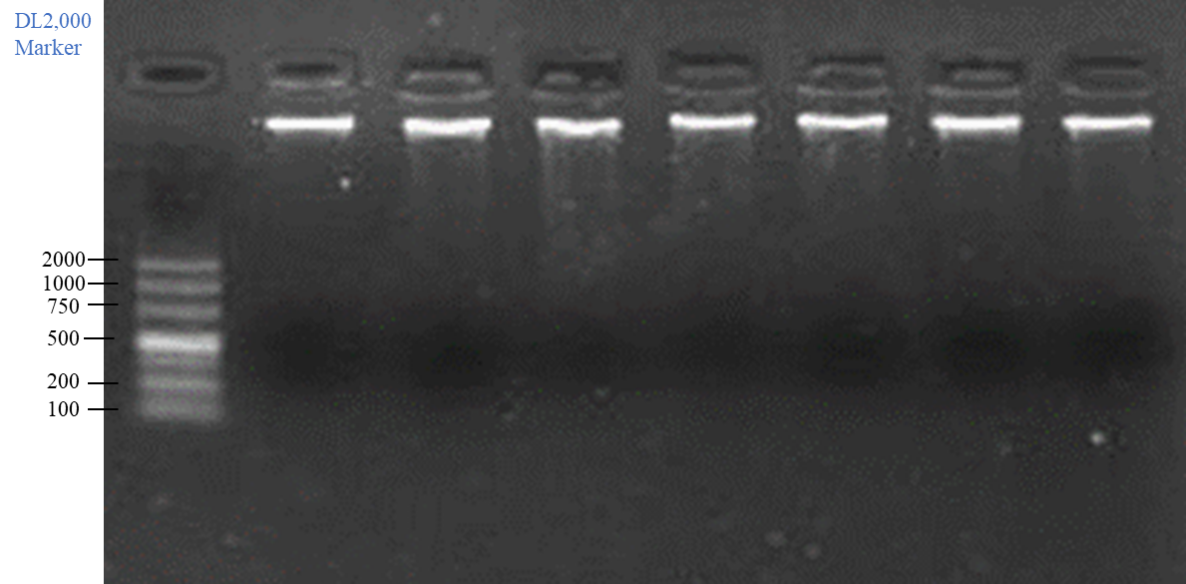

Supplement: R3 Supplementary Material File.docx [file LABT_A_2461176_SM7089.docx]
